# Complete mitochondrial DNA sequence of *Pseudogastromyzon changtingensis* (Osteichthyes: Gastromyzontidae)

**DOI:** 10.1080/23802359.2016.1250135

**Published:** 2016-12-23

**Authors:** Yang Yang, Xiaojing Song, Wenqiao Tang

**Affiliations:** a Laboratory of Ichthyology, Shanghai Ocean University, Shanghai, China;; b Shanghai Key Laboratory of Marine Animal Taxonomy and Evolution, Shanghai, China

**Keywords:** Mitochondrial genome, *Pseudogastromyzon changtingensis*, phylogenetic analysis, Gastromyzontidae

## Abstract

*Pseudogastromyzon changtingensis* belonging to the family Gastromyzontidae is a good model for phylogeny and zoogeography research. In the present paper, the sequenced mitochondrial genome of *P. changtingensis* is of 16573 bp in length, and encodes 13 typical protein-coding genes, two ribosomal RNA genes, 22 transfer RNA genes, and one control region. The result of phylogenetic analysis demonstrates that *P. changtingensis* is close to that of *P. fasciatus*.

The *Pseudogastromyzon changtingensis* belongs to the family Gastromyzontidae, which only lives in China and Southeast Asia. The distinguishing features of this family are pectoral and pelvic fins modified into sucker organs for clinging to objects in fast-flowing streams, and single unbranched anterior ray in pectoral and pelvic fins (Nelson et al. 2016). The species *P. changtingensis* distributes only in Beijiang and Xijiang river systems in China and is a good model for phylogeny and zoogeography research (Yue 2000). In this paper, the complete mitochondrial genome of *P. changtingensis* was determined. The sample of fish was collected from Ganjiang River (26°25′17.9″N, 116°30′44.8″E), Jiangxi province, China. The specimen is stored in the Laboratory of Ichthyology, Shanghai Ocean University, with accession number SLJXWY151023027. The sample DNA is available upon request.

The mitochondrial genome is typically a single circular chromosome in eukaryotes (Zhang et al. 2015). In the present study, the sequenced mitochondrial genome of *P. changtingensis* (GenBank accession number: KX669032) is of 16573 bp in length, and encodes 13 typical protein-coding genes, two ribosomal RNA genes, 22 transfer RNA genes (2 tRNA^leu^, 2 tRNA^ser^), and one control region (CR or D-loop). Most genes are encoded on the H strand, except for the ND6 gene and eight tRNA genes (tRNA^Gln^, tRNA^Ala^, tRNA^Asn^, tRNA^Cys^, tRNA^Tyr^, tRNA^Ser^, tRNA^Pro^, and tRNA^Glu^), which are encoded on the L strand. The nucleotide composition of mtDNA of *P. changtingensis* is A (29.45%), T (25.28%), C (28.55%), G (16.73%), which reflects that the percentage of A and T (54.72%) is higher than G and C (45.28%). The lengths of 22 tRNA range from 66 bp (tRNA^Cys^) to 76 bp (tRNA^Lys^). Apart from COI utilizing GTG, the rest of the 12 protein-coding genes start with the same initiation codon ATG. According to the result given by Mitoannotator (Iwasaki et al. 2013), there are seven protein-coding genes with the order of ND2, COII, ATP6, COIII, ND3, ND4, Cytb ending with incomplete stop codon (T-, T-, TA- TA-, T-, TA-, T-), while two genes (COI, ND5) use the stop codon TAA, and ND4L, ND1, ND6 use the stop codon TTA, TAG, TGA, respectively. The end of ATP8 overlaps with the beginning of ATP6 with a length of 10 bp, and there is another overlap between ND5 and ND6 with a length of 4 bp.

To investigate the phylogenetic relationship among the family Gastromyzontidae, we downloaded the mitochondrial genome sequences of 10 currently available species of Gastromyzontidae, including *Beaufortia kweichowensis* (KX060617), *B. szechuanensis* (KP716708), *Crossostoma lacustre* (AP010774), *Liniparhomaloptera disparis disparis* (AP013301), *Plesiomyzon baotingensis* (KF732713), *Pseudogastromyzon fasciatus* (KX101229), *Pseudogastromyzon myersi* (AP013300), *Sewellia lineolate* (AP011292), *Vanmanenia pingchowensis* (KP005457), and *Yaoshania pachychilus* (KT031050), together with African lungfish *Protopterus annectens* (NC018822) as outgroup species. The phylogenetic trees were constructed using MEGA6 (Tamura et al. 2013) for neighbour-joining, maximum-likelihood, and maximum parsimony methods. Tree topology was evaluated by 1000 bootstrap replicates. Different methods give the same tree topology, and the result demonstrates that *P. changtingensis* is close to that of *P. fasciatus* (Figure 1).

**Figure 1.**
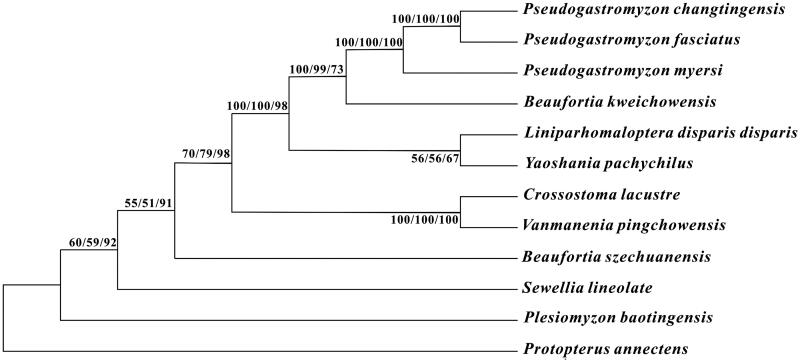
Phylogenetic tree of the family Gastromyzontidae, with African lungfish *P. annectens* as an outgroup. The topology of phylogenetic tree was inferred from neighbour-joining, maximum-likelihood, and maximum parsimony methods. Bootstrap supports for each analysis are indicated at the nodes.
